# The Role of Autophagy, Mitophagy and Lysosomal Functions in Modulating Bioenergetics and Survival in the Context of Redox and Proteotoxic Damage: Implications for Neurodegenerative Diseases

**DOI:** 10.14336/AD.2015.0820

**Published:** 2016-03-15

**Authors:** Matthew Redmann, Victor Darley-Usmar, Jianhua Zhang

**Affiliations:** 1 Center for Free Radical Biology,; 2 Department of Pathology, University of Alabama at Birmingham,; 3 Department of Veterans Affairs, Birmingham VA Medical Center, Birmingham, Alabama 35294, USA

**Keywords:** oxidative stress, reductive stress, mitochondrial dysfunction, prions, α-synuclein, neurodegenerative diseases

## Abstract

Redox and proteotoxic stress contributes to age-dependent accumulation of dysfunctional mitochondria and protein aggregates, and is associated with neurodegeneration. The free radical theory of aging inspired many studies using reactive species scavengers such as alpha-tocopherol, ascorbate and coenzyme Q to suppress the initiation of oxidative stress. However, clinical trials have had limited success in the treatment of neurodegenerative diseases. We ascribe this to the emerging literature which suggests that the oxidative stress hypothesis does not encompass the role of reactive species in cell signaling and therefore the interception with reactive species with antioxidant supplementation may result in disruption of redox signaling. In addition, the accumulation of redox modified proteins or organelles cannot be reversed by oxidant intercepting antioxidants and must then be removed by alternative mechanisms. We have proposed that autophagy serves this essential function in removing damaged or dysfunctional proteins and organelles thus preserving neuronal function and survival. In this review, we will highlight observations regarding the impact of autophagy regulation on cellular bioenergetics and survival in response to reactive species or reactive species generating compounds, and in response to proteotoxic stress.

As the population ages, the prevalence of debilitating age-related diseases increases leading to both significant decreases in the quality of life for the individual and an ever increasing economic burden on society as a whole. Redox and proteotoxic stress, metabolic imbalance, and mitochondrial dysfunction, have all been proposed to be associated with aging [1-6]. A common theme of these processes is that they all regulate autophagy, the process that degrades old, excessive or damaged macromolecules and organelles. Therefore, in response to stress, cell survival is dependent on optimal activation of autophagy. This is particularly important for terminally differentiated cells like neurons. Here we highlight some of the supporting evidence in the role of autophagy, mitophagy and lysosomal functions in modulating bioenergetics and survival in the context of redox and proteotoxic damage: implications for neurodegenerative diseases.

## Redox and proteotoxic stress in neuronal aging

Neurons are highly dependent on mitochondrial respiration for their function, including depolarization and homeostasis, and because of their non-dividing nature may have a higher propensity to accumulate oxidative damage, and thus the quality control of their organelles is important [7]. Mitochondrial dysfunction, oxidative and nitrative stress are pronounced in neurodegenerative diseases, and contribute to the initiation and progression of neurodegeneration [8-16]. Mitochondria can also both generate and be modified by reactive species. ROS and oxidative stress have also been implicated in the recruitment of specific mitochondrial autophagy proteins [17]. Furthermore, accumulation of oxidatively-modified and aggregated proteins, mitochondrial damage and loss of energy-generating capacity are prominent features in age-dependent neurodegenerative diseases [8]. For instance, the lipid peroxidation product 4-hydroxynonenal (HNE) accumulates as protein adducts in both Parkinson’s and Alzheimer’s disease brains [18-22].

Mitochondrial DNA mutations and electron transport chain deficits have been detected in Alzheimer’s and Parkinson’s diseases, with these deficits undoubtedly contributing to disease progression [8-16]. Experimental therapeutic approaches to reduce mitochondrial dysfunction by using a toxin-resistant complex I subunit, enhancing complex II activities, or stabilizing mitochondrial membrane potential [23-25] have been explored. Complex IV is important for mitochondrial respiration, and is sensitive to oxidative and nitrosative stress. Whether its expression serves to stimulate or inhibit signals for mitophagy and changes cellular resistance to oxidative stress are unknown. Translating these studies to successful clinical trials though, is a challenge [26]. Determining what controls mitochondrial fitness is important for a better understanding of neurodegeneration and arming us with the knowledge that may help design better therapeutic strategies. How cellular bioenergetics, oxidative stress and autophagic-lysosomal activities are cross regulated is also important. It is clear that mitochondrial activity and oxidative stress are intertwined as mitochondria are sensitive to ROS damage while simultaneously generating ROS for cell signaling. Indeed, inhibiting the mitochondria’s antioxidant defense mechanisms is deleterious to not only the mitochondria, but the cell as a whole, as best evidenced by SOD2 deletions or mutations [27]. Furthermore, inhibition of the mitochondrial glutathione transporter increases neuron sensitivity to oxidative and nitrosative stress [28]. The same degree of oxidative stress may then induce variable effects on bioenergetic programs depending on the maintenance of mitochondrial quality and coordination between biogenesis and mitophagy [29].

While uncontrolled levels of ROS are thought to be detrimental, and the accumulation of protein and DNA oxidation products and damaged mitochondria in aging animals has been taken as support for the free radical theory of aging [30,31,31,31], there has also been evidence that challenge this theory, with mild to moderate ROS elevation playing important roles in cell signaling, adaptation and lifespan extension. Manipulation of the enzymes responsible for generation and elimination of ROS does not directly support the idea that high ROS shortens and low ROS extends lifespan [6,32]. Programmed knockdown of mitochondrial complex I, III, IV and V activities may be associated with decreased metabolic rate and thereby contribute to longevity. For example, in worms, knockdown of cytochrome c oxidase-1 subunit Vb (COX4), by transgenic expression of an inverted repeat hairpin directed to COX4 gene in the intestine or neurons enhanced lifespan. COX4 knockdown did not confer resistance to paraquat-induced oxidative stress, UV damage, or heat stress, while inducing the mitochondria-specific unfolded protein response (UPR^mito^) [33]. Mutation of the Rieske iron sulfur protein or a subunit of complex I of the mitochondrial electron transport chain led to increased lifespan with an associated elevated mitochondrial ROS [34], and an Apaf1 and caspase-9 dependent alteration of transcriptomes in the cell is responsible for the enhanced longevity [35]. Similarly, mutation of a mitochondrial hydroxylase required for ubiquinone production also led to enhanced lifespan and with associated increase of ROS and HIF-1-dependent gene expression [36]. Although in *C.elegansdaf-2* mutation led to decreased mitochondrial activity and decreased ROS levels and increased lifespan, acute impairment of *daf-2* in adult worms led to transient increase of ROS, which induced adaptive response and is required for enhanced life span by *daf-2* impairment[37]. Further supporting a lack of direct relationship between ROS levels and aging, knockdown of the mitochondrial SOD extended lifespan in worms [38], and the extension of lifespan by overexpression of SOD-1 is not associated with decreased lipid oxidation or glycation, but associated with increased protein oxidation and ER stress and is dependent on the transcription factor FoxO, IRE-1 and XBP-1 [39]. Knockout of all 5 superoxide dismutases (SODs) are not essential for normal lifespan despite markedly increased sensitivity to multiple stresses in worms[40]. However, in marked contrast to worms, SOD2 or SOD1 knockout in mice develop cardiomyopathy, neurodegeneration, or neuromuscular junction degeneration, respectively, and decreased lifespan [41-46], while neither SOD1 nor SOD2 overexpression in mice extends lifespan [47]. Although deficiency in proofreading activities of PolG of mitochondrial DNA led to increased somatic mtDNA mutations and decreased lifespan [48], studies in the flies indicated that oxidative stress is not a major contributor to somatic mitochondrial DNA mutations [49].

**Figure 1.**
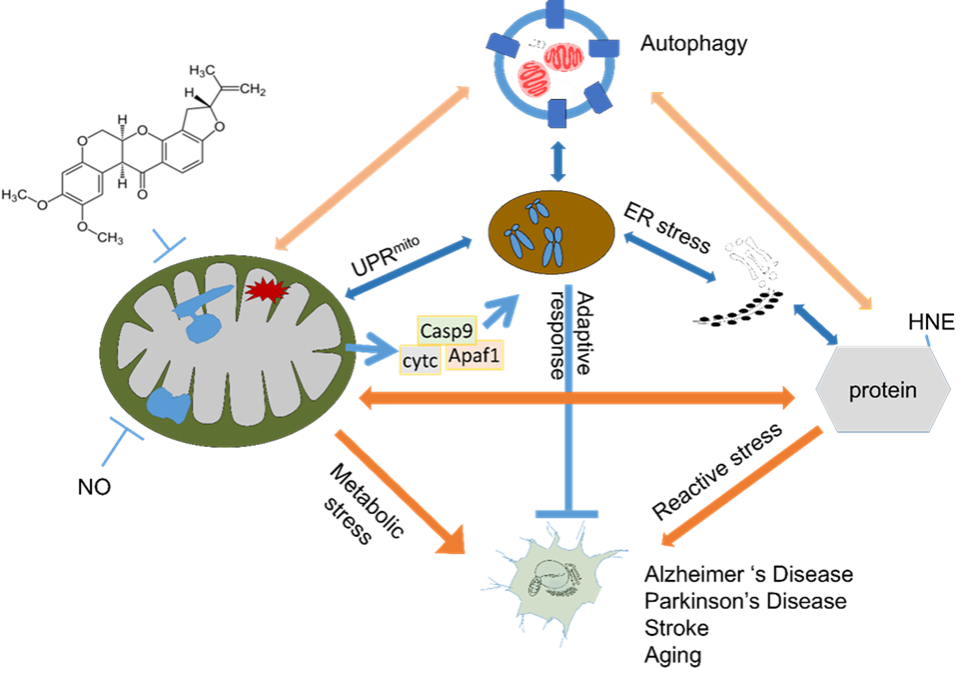
**Autophagy serves as an essential neuroprotective pathway in response to mitochondrial dysfunction and oxidative stress**. In neurodegenerative diseases, AD, PD, and stroke, mitochondrial dysfunction accumulates due to aging, genetic abnormalities, environmental damage (such as pesticides), or neuroinflammation (which induces excessive production of nitric oxide, among others), resulting in decreased oxidative phosphorylation, and accumulation of mtDNA damage. There are also increases in protein damage, including protein oxidation and formation of HNE-protein adducts. Whether absolute levels of ROS are directly correlated with aging process is debatable. Emerging evidence indicated that transient or moderate ROS elevation may trigger response in ER stress and mitochondrial unfolded protein response pathways, as well as adaptations mediated by HIF, NRF2 and other transcription factor-regulated mechanisms (such as Apaf1 and Caspase-9 dependent mitochondria to nuclear signaling). Therefore, a systemic decrease of ROS is unlikely to be the best approach to delay aging and age related neurodegeneration. Clearance of damaged proteins and organelles are dependent on the autophagy process, which involve double membrane vesicles encircling these damaged intracellular materials and sending them to be degraded. It has been hypothesized that dysfunction of autophagy promotes neurodegeneration and enhancement of autophagy may be neuroprotective.

Taken together, these data cannot be easily reconciled with either the oxidative stress hypothesis or the free radical theory of aging in their simplest manifestations. However, emerging evidence in the redox biology field places these findings in a different context. It is now clear that a critical role of intracellular antioxidants, such as glutathione or superoxide dismutase, is to maintain the integrity of redox signaling domains and that reductive stress can be as detrimental as oxidative stress. It has also been shown that mitochondrial ROS (superoxide or hydrogen peroxide) can be generated at multiple sites within the organelle and these are regulated by substrate supply and are not necessarily equivalent with respect to their downstream signaling effects [50-52]. The impact of manipulating these pathways can then only be interpreted in the context of their interaction with metabolism and cell signaling. In this regard, enhanced autophagic activity may provide additional survival signals or mechanisms for the cell to manage either transient or prolonged increases in oxidative damage to proteins as well as damage that occurs independently of ROS in the context of aging and longevity (Figure 1).

## The role of autophagy and mitophagy in lifespan and neuronal aging

The importance of autophagy in aging is supported by observations that yeast, *C.elegans* and flies with impaired autophagy have decreased lifespan. This contributes to the notion that that autophagy plays an important role in the aging [53-55]. Physiologically, autophagy deficient skeletal muscles and pancreatic β cells have dysmorphic mitochondria and defective oxidative phosphorylation [56]. PINK1 knockout mice exhibit mitochondrial dysfunction in cultured primary cortical neurons as well as the striatum, liver, and brain[57,58]. Furthermore, pharmacologic or genetic manipulations that increase life span in model organisms often stimulate autophagy [59-66]. For example, inhibition of mTOR by rapamycin, which enhances autophagy, extends health span and lifespan in model organisms [67]. The mechanisms of the effect of rapamycin are pleiotropic, including inhibition of protein synthesis, alteration of transcriptomes, modulation of inflammation, and improvement of cerebral blood flow, in addition to regulation of autophagy, mitophagy and thereby mitochondrial function [59,64,68-80]. Taken together, these examples provide strong support for the concept that autophagy plays a critical role in maintaining a normal lifespan and healthy neuronal aging, and that its decline is inexorably tied to age related pathologies.

Degradation of dysfunctional mitochondria is carried out by the autophagy-lysosomal pathway. A deficiency in the autophagy protein Atg7 has been shown to cause mitochondrial dysfunction, both in isolated mitochondria from skeletal muscle and in cultured embryonic fibroblasts [56]. Furthermore, intracellular ROS levels were increased in autophagy gene Atg7 knockout cells [56]. Parkin knockout results in aberrant mitochondrial morphology and activities [81]. Many lysosomal diseases result in deficient autophagic degradation of cellular materials. The alteration of mitochondrial morphology and decreased respiratory chain activity varies in both extent and characteristics in tissues and isolated mitochondria among these diseases [82]. The lysosomotropic agent chloroquine or the lysosomal protease cathepsin D inhibitor pepstatin A have both been shown to increase the formation of reactive species [83-87]. How *in vivo* blockade of autophagy impacts mitochondrial morphology and specific respiratory chain activities is not well understood. Understanding this relationship will be crucial in identifying specific targets for therapeutic modulation that are particularly sensitive to autophagic stress associated with neurodegenerative diseases.

Healthy neurons exhibit a significant basal level of autophagic flux, and such flux can be easily attenuated by perturbations of lysosomal activities seen in various lysosomal storage diseases [88-92], and autophagy-lysosomal degradation system declines with age [93-95]. Enhancing autophagosomal activity without enhancing lysosomal enzyme activities may not be effective. Further, enhancing overall macroautophagy, as in treatments that stimulate the cell to initiate starvation responses, may be detrimental because of a risk of eliminating normal, but essential proteins. Enhancing lysosomal function may increase the efficiency of flux and degradation of already damaged autophagosome sequestered proteins and organelles, thus preserving selectivity for toxic or dysfunctional material, and contributing to neuroprotection. Improving lysosomal biogenesis, increasing specific hydrolases, or decreasing lysosomal protease inhibitors have all been explored as neuroprotective strategies [96-98]. Here we highlight some of the evidence that autophagy may serve as an antioxidant and anti-proteotoxicity pathway and provide a beneficial impact on neuronal bioenergetic health and survival (Figure 1).

## The role of autophagy in response to oxidative and nitrosative stress, as well as mitochondrial inhibition in neuronal cells

Mitochondria are targets and generators of oxidative stress and their dysfunction is prominent in age dependent diseases. Therefore assessing mitochondrial function in response to genetic deficits and/or oxidative stress is important. We and others have optimized the technique of using the Seahorse extracellular flux analyzer to measure basal, complex V-linked ATP generation, as well as uncoupling induced maximal oxygen consumption from the mitochondria [99]. Furthermore, using plasma membrane permeabilization agents and supplementing substrates for different mitochondrial respiratory chain components, complexes I, II, IV and V activities can be individually measured in a high throughput manner[100]. These measurements coupled with mtDNA copy number and a mtDNA damage assay [101-105], mitochondrial reactive oxygen species measurements[106], protein level and post-translational modification analyses [107], have allowed us to demonstrate an important role of autophagy in response to starvation, unfolded protein response, and oxidative stress in diverse cell types, including mouse embryonic fibroblast, endothelial cells, β islet cells and breast cancer cells[108-112]. These observations suggest that autophagy is important for redox homeostasis, mitochondrial quality control, cell proliferation and survival [29,113,114]. Furthermore, we and others have found that autophagy and lysosomal gene expression may be coordinately regulated at transcriptional and post-transcriptional levels, and that genetic mutations of autophagy genes lead to abnormal cell and tissue homeostasis [115,116,116-118]. Future studies regarding how autophagy regulation at both transcriptional and post-translational levels in response to diverse stimuli in a wide range of cellular contexts are urgently needed.

Post-mitotic cells do not have the capacity to divide and dilute toxic cellular constituents, and thus may be more susceptible to accumulation of oxidatively damaged proteins. We have investigated the bioenergetic difference between proliferating neuroblastoma and differentiated neuroblastoma SH-SY5Y cells. We found that differentiated SH-SY5Y cells exhibited increased mitochondrial membrane potential as assessed by JC-1 red/green fluorescence, increased maximal oxygen consumption rate and decreased extracellular acidification rate, while basal oxygen consumption rate, mitochondrial DNA copy number and citrate synthase activities normalized to total protein remain the same[119]. Consistent with a higher reserve bioenergetic capacity, the differentiated cells exhibit higher resistance to mitochondrial inhibition and cell death induced by lipid peroxidation product HNE or intracellular ROS generator, 2,3-dimethoxy-1,4-napthoquinone (DMNQ)[119]. We found that in the differentiated SH-SY5Y cells, different neurotoxins have significantly different impact on cellular bioenergetics at similar toxicity doses, indicating distinct mechanisms of action which cannot be explained by simply inhibiting complex I[99,119,120]. Glycolytic activities play an important role in supporting basal and HNE-induced autophagy as well[121].

**Figure 2.**
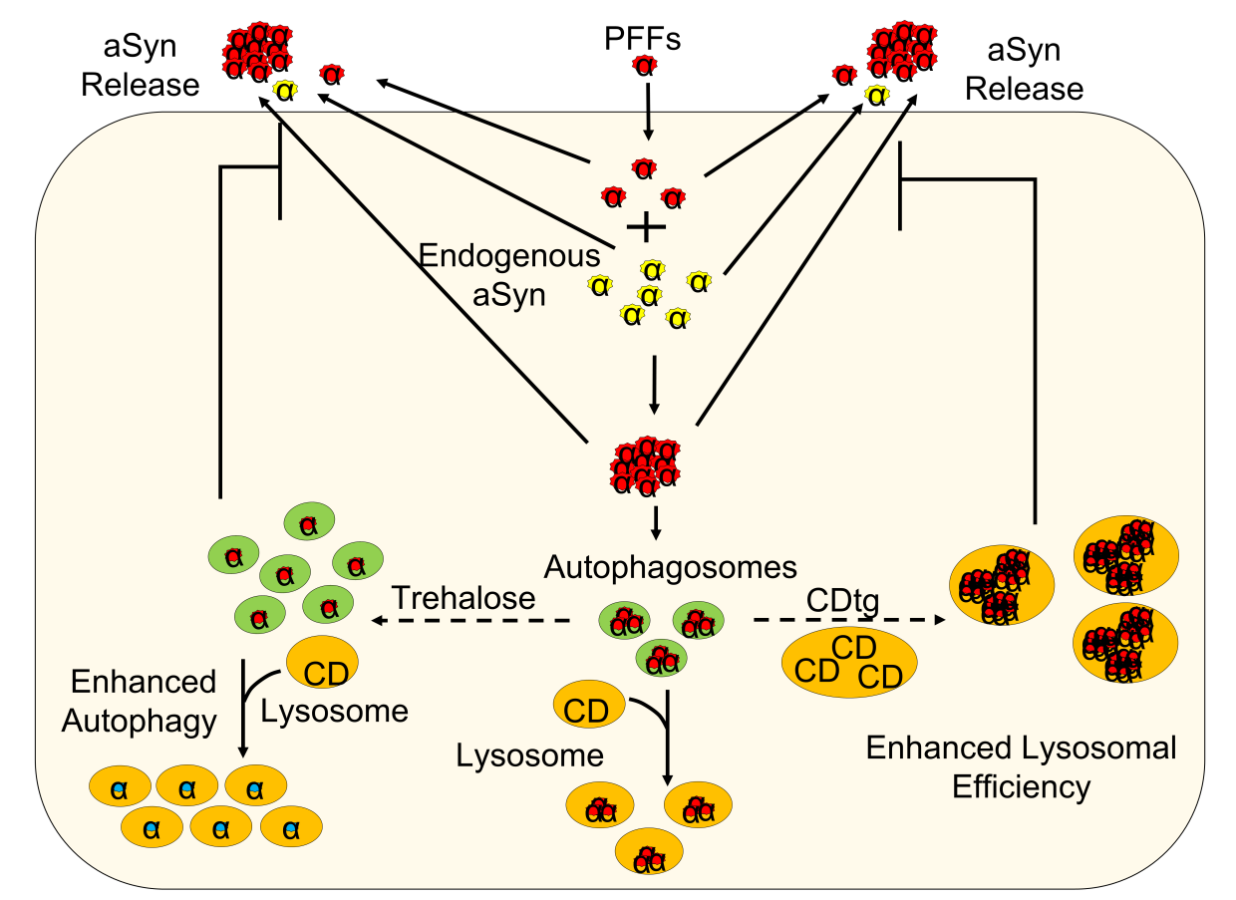
**Autophagy may be used to attenuate α-synuclein secretion and inter-cellular propagation**. α-synuclein fibrils (PFF) (red circles) recruit endogenous α-synuclein (aSyn) (yellow circles) to form aggregates and induce neuron death. Aggregates can also be released and propagate to neighboring cells and further pathological damage to the brain. Enhanced lysosomal efficiency/hydrolytic capacity through increased Cathepsin D or enhanced autophagosome production through trehalose treatment may promote the sequestering and degradation of toxic α-synuclein species.

Rotenone has been shown to be one of the pesticides linked to increased risk for Parkinson’s disease due to its potent inhibitory effects on mitochondrial complex I [122-125]. Furthermore, injection of rotenone into rats and oral administration to mice induced α-synuclein accumulation and dopaminergic neurodegeneration [126,127]. We found that in live rat primary cortical neurons, rotenone as low as 10 nM induced immediate mitochondrial respiratory inhibition. Although autophagic flux is inhibited by 10 nM rotenone, mitophagy is increased. Cell death is attenuated by the autophagy stimulator rapamycin and exacerbated by the autophagy inhibitor 3-methyladenine (3-MA). With regard to neuronal bioenergetics, reprogramming of mitochondrial substrate usage associated with both complex I and complex II activities occurred in response to rapamycin [128]. In comparison, neuronal response to nitric oxide is highly dependent on oxygen tension, as nitric oxide donors significantly inhibited basal oxygen consumption in hypoxia (1% oxygen) but not in normoxia (20% oxygen). The recovery of mitochondrial respiration in reoxygenation is incomplete, consistent with irreversible damage due to the exposure of neurons to nitric oxide and/or superoxide generated from the mitochondrion. The recovery is further attenuated in the presence of the autophagy inhibitor 3-MA, further demonstrating an essential role of autophagy in preserving neuronal bioenergetics [110]. Taken together, autophagy plays an important role in mitochondrial quality control and neuronal survival (Figure 1).

## The role of autophagy in response to proteotoxicity

One of the prototypical neurodegenerative diseases, Parkinson’s disease, is characterized by the progressive and currently unstoppable loss of dopaminergic neurons in the substantia nigra and many other brain regions (18). One dominant defining feature shared among Parkinson’s and other neuropathologies, including dementia with Lewy bodies and multiple systems atrophy, is the accumulation of α-synuclein(19-21). One critical issue for α-synuclein pathology is that it can leave the cell and seed aggregations in neighboring cells, acting in a prion-like fashion (12). The spread of α-synuclein is worsened by autophagic decline, a pattern observed in PD models[129]. This presents an important mechanism for disease progression, and warrants serious consideration for therapeutic development. How α-synuclein leaves the cell and seeds aggregations elsewhere is still unclear, but exosomal or exocytosis-mediated mechanisms, along with direct diffusion through plasma membranes, have been proposed (22-24). Notably and central to our research focus is the observation that a decrease in lysosomal function promotes the release of α-synuclein from the cell, and that α-synuclein can seed further aggregation in a new population of cells (24).

Many α-synuclein overexpression models have been generated in rodents, each tests a specific aspect of neurodegeneration, although none fully recapitulate human disease pathogenesis (25-29). Recent work demonstrated that preformed α-synuclein fibrils can be taken up into neurons and seed the formation of endogenous α-synuclein aggregates that resemble many aspects of Lewy body and Lewy neurite pathologies, including forming insoluble, ubiquitinated and phosphorylated high molecular weight species that further amplify disease pathogenesis (Figure 2) (12, 16, 17). Major protein degradation pathways in neurons include the proteasome, chaperone-mediated autophagy and macroautophagy. For two of these pathways the lysosome is the rate-limiting step in neurons (13). Increasing autophagy initiation without increasing lysosomal mediated degradation may not be effective in degrading α-synuclein and attenuating its propagation. Human patients who lack functional Cathepsin D whether by protein truncation, misfolding, or depletion results in congenital neuronal ceroid-lipofuscinosis (NCL), a disease that results in rapid neurodegeneration and death within hours to weeks after birth (46). Cathepsin D knockout mice were found to exhibit a similar phenotype, ultimately leading to death at approximately p25 (47). Chemical inhibition of proteasomal function does not consistently produce α-synuclein accumulation in animal models (30, 31), whereas we and others have found that autophagy deficiency in cathepsin D mutant humans and animal models resulted in significant aSyn accumulation (1, 14, 32, 33). Furthermore, we have previously shown that overexpression of wildtype Cathepsin D, but not cathepsin B, L, or mutant Cathepsin D, decreased α-synuclein toxicity in worms *in vivo* and mammalian cells *in vitro* (1). We also found that in addition to Cathepsin D, Cathepsins B and L also protects against mutant huntingtin-induced neuron death, while inhibition of autophagy-lysosomal functions by 3-MA, E64d, Pepstatin A exacerbated mutant huntingtin-induced neuron death [130]. These observations indicate that lysosomal function and efficiency are crucial in normal cellular function, and that its enhancement may be beneficial in promoting the removal of toxic long-lived proteins.

One of the major regulators of macroautophagy is the mammalian target of rapamycin (mTOR). Activation of autophagy by rapamycin and its derivatives, via inhibition of mTOR, has shown benefits in cell and animal models as treatments for neurodegenerative diseases (35), however, mTOR inhibition also leads to decreased protein synthesis; furthermore, side effects of rapamycin in humans limit its use, especially over the long term. Furthermore, rapamycin fails to clear intracellular aggregates that are resultant from preformed α-synuclein fibril exposure and ultimately exacerbating cell death. [131]. mTOR independent autophagy activators have also been found, one of them being trehalose, which is considered safe for human use at doses up to 50g in drinking water (36). Although no epidemiology studies have been performed to correlate its consumption with PD incidence, trehalose has been shown to activate autophagy and decrease α-synuclein aggregates in PC12 cells (37), decrease Tau accumulation in mice (38), and decrease neurodegeneration in amyotrophic lateral sclerosis (39), Huntington’s (40), and Alzheimer’s (41) disease models. Trehalose has been shown to be safe with no use limits in Korea and Taiwan, up to 5% in food in UK, and in 2000 the US FDA gave a letter of no objection to a GRAS Notice for human consumption (42). In drinking water at a concentration of 1-5%, at several age groups from 3 weeks to 4 months, trehalose has been shown to be beneficial in increasing autophagy, while decreasing the accumulation of Tau and dopaminergic neuron death, and improving behavioral performance in *Parkin* knockout mice overexpressing human mutated Tau protein (38-41). Although the exact mechanism of its action is unclear, one potential mechanism may be activation of the transcription factor TFEB which activates more than 30 autophagy and lysosomal genes; however other mechanisms may also contribute to its action (43-45). Modulation of TFEB alone has also shown promise, where its genetic overexpression of pharmacologic induction has been shown to promote α-synuclein degradation [132,133]. Nonetheless, increasing the overall lysosomal numbers may or may not be effective in attenuating α-synuclein aggregate formation after neurons are exposed to α-synuclein fibrils, since it has been shown that lysosomal cathepsin B may promote aggregate formation [134].

In addition to aberrant accumulation of toxic protein species, mitochondrial dysfunction has also been reported to be in widespread brain areas, including both the substantia nigra and the cortex in Parkinson’s disease brains [9,135-137]. How mitochondrial dysfunction is produced is unclear. Environmental toxins such as rotenone and paraquat that damage mitochondria have been shown to induce dopaminergic degeneration in animal models and increase Parkinson’s disease risk in humans [122-124]. Importantly, α-synuclein can target to mitochondria via an N-terminal cryptic sequence [138], decrease complex I activity and increase the production of reactive oxygen species [138-146]. Prior studies investigated the effects of bypassing complex I blockade via supplying a toxin-resistant subunit, enhancing complex II activities, stabilizing mitochondrial membrane potential [23-25], or enhancing reactive oxygen species clearance [26,147-149] in various animal models. However, clinical trials based on these direct approaches have had limited success. The limitations include the possibilities of perturbing cellular bioenergetics and redox signaling, and the inability to reverse already propagated damage to proteins and organelles. An alternative approach to improving bioenergetic function in response to α-synuclein accumulation may be to enhance autophagy-lysosomal activities [150].

## Conclusion

Reactive damage to proteins and organelles contributes to age-dependent accumulation of dysfunctional mitochondria and protein aggregates and is associated with neurodegeneration. Autophagy has been shown to play a protective role in cellular response to reactive oxygen and nitrogen species as well as toxic proteins. Further investigation of the mechanistic and regulatory elements of autophagy and their respective contributions as a function of time on age related disease pathogenesis will provide important insights into designing therapeutics to stall disease progression.
